# The paradox of highly effective sofosbuvir-based combination therapy despite slow viral decline: can we still rely on viral kinetics?

**DOI:** 10.1038/s41598-017-09776-z

**Published:** 2017-08-31

**Authors:** Thi Huyen Tram Nguyen, Jérémie Guedj, Susan L. Uprichard, Anita Kohli, Shyam Kottilil, Alan S. Perelson

**Affiliations:** 1 IAME, UMR 1137, INSERM, F-75018 Paris, France; Univ Paris Diderot, Sorbonne Paris Cité, F-75018 Paris, France; 2 Hopital Henri Mondor, Université Paris-Est, Creteil, France; 3 Department of Medicine, Loyola University Medical Center, Maywood, Illinois USA; 4 Dignity Health, St. Joseph’s Hospital, Phoenix, Arizona USA; 5 Laboratory of Immunoregulation, NIAID, NIH, Bethesda, MD USA; 6 Theoretical Biology and Biophysics Group, Los Alamos National Laboratory, Los Alamos, NM USA

## Abstract

High sustained virologic response (SVR) rates have been observed after 6 weeks of anti-HCV treatment using sofosbuvir, ledipasvir and a non-nucleoside polymerase-inhibitor (GS-9669) or a protease-inhibitor (GS-9451) and after 12 weeks with sofosbuvir + ledipasvir. Here we analyze the viral kinetics observed during these treatments to decipher the origin of the rapid cure and to evaluate the possibility of further reducing treatment duration. We found that viral kinetics were surprisingly slow in all treatment groups and could not reproduce the high SVR rates observed. Based on experimental results suggesting that NS5A- or protease-inhibitors can generate non-infectious virus, we incorporated this effect into a mathematical model. We found that to predict observed SVR rates it was necessary to assume that ledipasvir, GS-9669 and GS-9451 rapidly reduce virus infectivity. We predicted with this model that 4 weeks of triple therapy could be sufficient to achieve SVR in patients with undetectable viremia at week 1, but would be suboptimal in general. In conclusion, the rapid cure rate achieved with these combinations is largely disconnected from viral loads measured during treatment. A model assuming that rapid cure is due to a drug effect of generating non-infectious virus could be a basis for future response guided therapy.

## Introduction

Chronic infection with hepatitis C virus (HCV) is a leading cause of advanced liver disease. In the past few years, the landscape of anti-HCV therapy has changed due to the development and commercialization of several direct-acting antiviral agents (DAAs), allowing rates of sustained virologic response (SVR), i.e. viral eradication, to increase from about 50% in 2010 to more than 90% nowadays^1^. In parallel the duration of treatment has been dramatically reduced, going from 48 to 12 weeks for most patients^2, 3^. Several studies demonstrated that the treatment duration could be even shorter in naïve or non-cirrhotic patients when combining two or three DAAs. For instance, in the ION-3 phase 3 study evaluating the combination of sofosbuvir and ledipasvir (SOF + LDV) in 647 HCV genotype 1 treatment naïve patients, 8 weeks of treatment was significantly non-inferior to 12 weeks of treatment, with SVR rates of 94% and 95%, respectively^4^. However, it is unlikely that this combination will allow for shorter treatment duration as a relapse rate of 30%, albeit on a small sample size (N = 25), was found in patients treated for only 6 weeks^5^. In the quest for shorter treatment duration, the SYNERGY trial (ClinicalTrials.gov, number NCT01805882) added a third antiviral, either a non-nucleoside polymerase inhibitor (GS-9669) or a protease inhibitor (GS-9451), on top of SOF + LDV. Although the number of patients was limited (N = 40), both combinations showed SVR rates of 95% after only 6 weeks of treatment^6^. This result is probably not limited to this combination and a SVR rate of 87% (N = 26/30) was also found with another SOF-containing triple therapy^7^. Whether triple therapy can achieve similarly high SVR rates after only 4 weeks of treatment is still unclear but results reported in a small number of patients (N = 25) with these combinations led to much lower SVR rates (40%), suggesting that 6 weeks was probably a minimal duration for many patient populations^8^.

Mathematical modeling of HCV kinetics has provided important insights into the HCV life cycle as well as the effectiveness and the mechanisms of action of different anti-HCV agents. With the rapid development of new DAAs, novel viral kinetic models have been developed to explain phenomenon observed with these new treatments, such as emergence of resistance, or to incorporate new mechanisms of action of HCV drugs such as blocking viral replication or assembly/secretion^9^. Recently, results of an *in vitro* experiment suggest that some DAAs, in particular NS5A and protease inhibitors, significantly affect viral infectivity and that infectious titer declines much more rapidly than extracellular viral load in response to these treatments^10^. This mode of action has not been previously integrated into viral kinetic models.

Since the end of the 1990’s, the monitoring of HCV RNA after treatment initiation has played a critical role in defining guidelines to tailor treatment duration^11^. Recently, a proof-of-concept trial was conducted in HCV patients to evaluate the possibility of using on-treatment HCV RNA levels to define the duration of triple DAA-based treatment and the results were promising^12^. In that study involving Chinese patients all infected with HCV genotype 1b without cirrhosis, all 18 patients who achieved a viral load <500 IU/mL by day 2 with triple DAA regimens were cured after only 3 weeks of treatment^12^. This suggests (but does not demonstrate) that viral kinetic models could be relevant to optimize therapy or to identify patients eligible for therapy as short 2–4 weeks.

Here we analyzed the viral kinetics observed during treatments with SOF + LDV + GS-9669/GS-9451 for 6 weeks, SOF + LDV for 12 weeks (Synergy trial) and SOF + ribavirin (RBV) for 24 weeks (SPARE trial: ClinicalTrials.gov, number NCT01441180) using mathematical modeling. By comparing the kinetics observed with these different treatment strategies we aim to decipher the biological origin of the rapid treatment response in short duration treatment and to evaluate if the *in vitro* finding that NS5A and protease inhibitors can reduce infectious virus is consistent with *in vivo* observations. We also evaluate whether the success of triple therapies could be predicted and whether it is possible to identify patients eligible for shorter treatment using a viral kinetic modeling approach.

## Results

### Viral kinetics description

**Figure 1:**
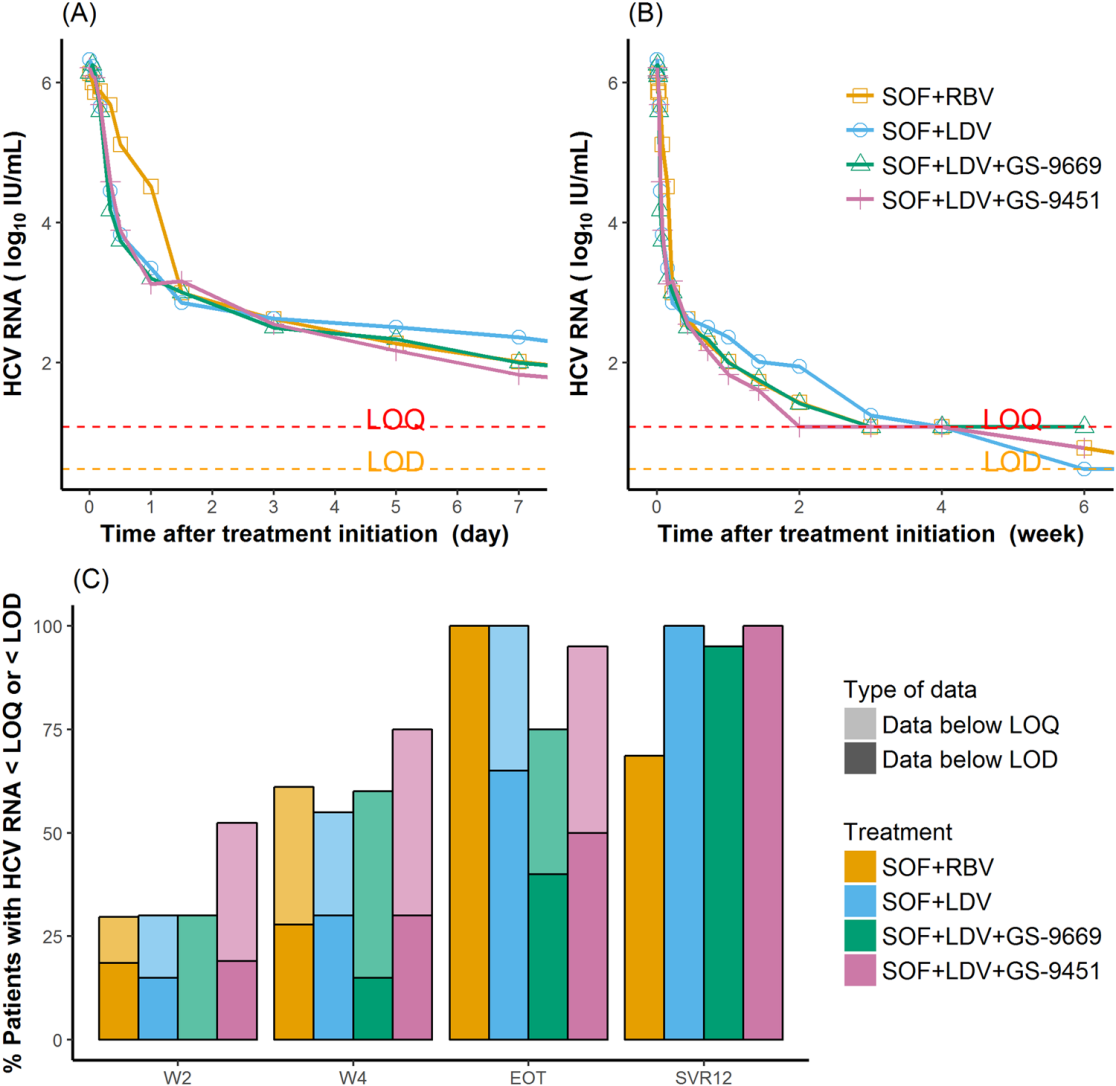
Observed viral kinetics and virologic responses in the SYNERGY and SPARE trials. Upper row: observed median viral load versus time during the first week of treatment (left panel) and during 6 weeks of treatment (right panel). Lower row:proportion of data below the limit of quantification (LOQ) and/or the limit of detection (LOD) during treatment. In these trials LOQ = 12 IU/mL and LOD = 3 IU/mL.

The early median viral decline in the different treatment groups suggests that treatment regimen does not have a strong influence on viral load levels after day 3 (Fig. 1A,B). At the end of treatment (EOT), all patients treated with SOF + RBV had HCV RNA below the limit of detection (LOD), whereas in the SYNERGY trial this proportion was only 65% (13/20), 40% (8/20) and 50% (10/20) in patients treated with SOF + LDV, SOF + LDV + GS-9669 and GS-9451, respectively. Moreover, 8 patients in the 6 week treatment groups had HCV RNA above the limit of quantification at EOT.

### Viral kinetic parameter estimates

**Figure 2:**
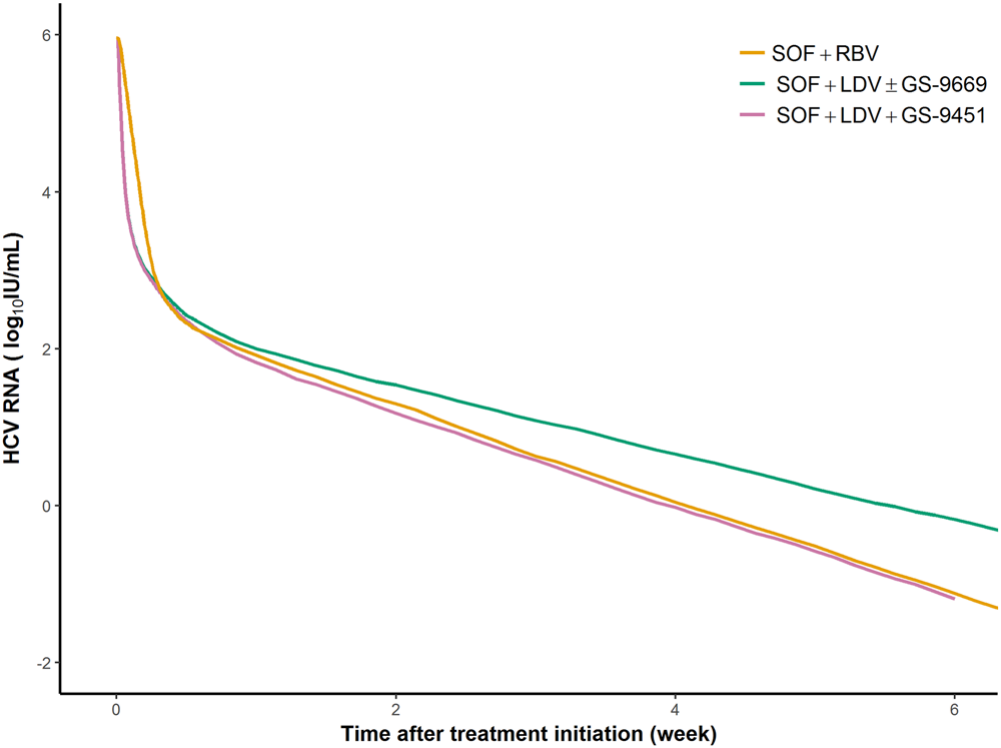
Median predicted total viral load (evaluated on 1000 simulations) according to treatment group. Because there was no difference in viral kinetic parameters in patients receiving SOF + LDV or SOF + LDV + GS-9669 (see Table 1), the two curves overlap.

Consistent with these observations, viral kinetic parameters obtained by fitting a multiscale model of HCV kinetics under therapy, Eq. (1) in Methods, did not show great differences across treatment groups (Table 1). The rapid decline in the first hours of treatment in patients treated with SOF + LDV containing regimes was explained in the model by a large effect in blocking viral assembly/secretion (ε_s_ = 0.997). However, no difference in blocking assembly/secretion was found across the three treatment groups, suggesting that LDV was the main driving force for this blocking, with minimal additional effect of the non-nucleoside polymerase inhibitor or the protease inhibitor. The treatment effect in blocking viral RNA production, *ε*_*α*_, was lower in SOF + LDV ± GS-9669/GS-9451 compared to SOF + RBV (ε_α_ = 0.98 vs 0.9996, p < 10^−10^). The loss rate of infected cells, δ, was larger in patients treated with SOF + RBV or SOF + LDV + GS-9451 than in patients treated with SOF + LDV ± GS-9669 (median δ of 0.2 and 0.14 day^−1^, respectively, p = 0.0025). The predicted viral kinetics in different treatment groups are shown in Fig. 2.

**Table 1:** Population parameter estimates obtained by fitting the multiscale model (Eq. 1)^35^ to total viral load kinetic data in the SPARE and SYNERGY trials until the end of treatment. Mean population parameter represents the typical value of a parameter in the studied population. The variability of each parameter is shown in the last column. We assumed a log-normal distribution for all parameters except ε_α_ and ε_s_, which were assumed to follow logit-normal distribution. RSE: relative standard error in percentage. When a parameter was fixed to values found in literature and was not estimated, the RSE could not be provided and this is indicated by “−”.

	Mean population parameter (RSE (%))	Covariate testing	Variability (RSE (%))
T_lag_	0. 071 (8)		0 (−)
log_10_(V_0_)	5. 96 (1)		0.133 (4)
c	14.4 (12)		0.919 (11)
δ_SOF+LDV±GS-9669_	0.141 (9)	p = 0.0025	0.608 (4)
δ_SOF+RBV or SOF+LDV+GS-9451_	0.198 (7)		
α	40 (−)		0 (−)
ρ	4.44 (9)		0.75 (9)
μ	0.87 (9)		0 (−)
ε_α_ __SOF+RBV_	0.9996 (2)	p < 10^−10^	1.02 (9)
ε_α _SOF+LDV±DAA_	0.98 (5)		
ε_s_ SOF+LDV±DAA_	0.997 (3)		0.852 (13)
κ	1 (−)		0 (−)

### The original multiscale model cannot predict SVR rates with SOF + LDV based therapy

We used the estimated viral kinetic parameters to predict the SVR rate from the predicted total viral load using a “cure boundary” approach (see Methods) for various treatment durations (Table S2). The predicted SVR rate after 24 weeks of SOF + RBV was 90%, i.e., higher than observed in reality (48–68% depending on the RBV dose). In the SYNERGY trial the SVR rates were largely underestimated with predicted SVR rates of 34% vs 100% (observed) for 12-week treatment with SOF + LDV, 6% vs 95% (observed) for a 6-week treatment with SOF + LDV + GS-9669 and 16% vs 100% (observed) for a 6-week treatment with SOF + LDV + GS-9451. This shows that the high SVR rates observed in the SYNERGY trial cannot be predicted with this method.

### The extended multiscale model including non-infectious virus can explain the paradox of the slow viral decline in patients treated with SOF + LDV based therapy

The simplest explanation to reconcile the high SVR rates observed in the SYNERGY trial in spite of a slow viral decline is that most of the HCV RNA measured is not contained in infectious virus particles. To explore this possibility, we extended the multiscale model to account for infectious virus, *V*_I_, and non-infectious virus, *V*_NI_, such that the total HCV RNA measured is equal to *V*_I_ + *V*_NI_. In this model, we assume that after treatment begins ${p}_{I}(t)={p}_{0}\ast {e}^{-\lambda t}$ is the time-dependent probability for a plus strand RNA to be secreted as an infectious virus particle, *p*_0_ is a constant (e.g. the pre-treatment proportion of infectious virus in the blood) and λ is a parameter measuring the progressive decrease in the proportion of infectious virus generated under treatment. If λ = 0, then the fraction of infectious virus produced under therapy is constant. In the Supplemental Material we call this proportion *p*_I_ and reserve *p*_0_ for the constant pre-treatment fraction of infectious virus. Because drug therapy may cause damage to the intracellular viral replication machinery that accumulates in time, we also consider the case in which λ > 0, i.e., we allow this fraction to decrease in time. We use an exponential model for simplicity and because it would result from a process where the loss of replication fidelity occurred at a constant rate. Further, we assume the damage that occurs is irreversible, so when therapy is stopped *p*_I_ does not return to *p*_0_. While the model does not specify why treatment induces a loss of infectivity, it could be due to defective viral RNA(vRNA), packaging vRNA in exosomes or defective virion assembly.

**Figure 3:**
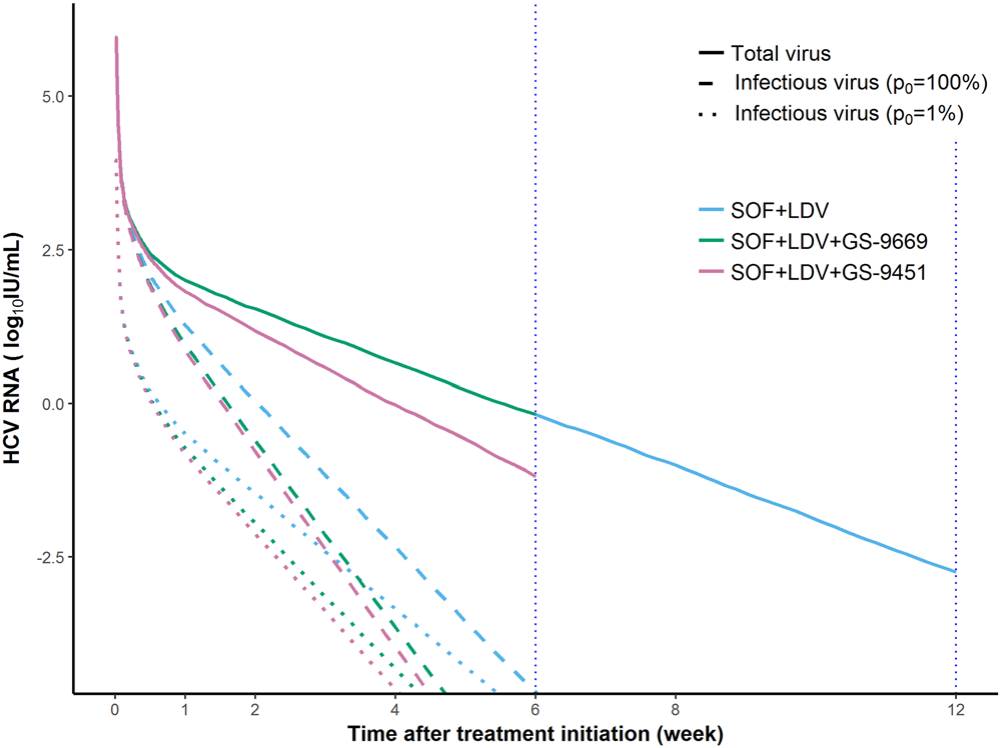
Median predicted total and infectious virus, assuming that a 100% or 1% of virus is infectious before treatment initiation.

If we assume that 1% of virus is infectious at the beginning of treatment, λ needs to be equal to 0.17, 0.25 and 0.22 day^−1^ to match the 95% SVR rates observed with SOF + LDV (8 weeks), SOF + LDV + GS-9669 (6 weeks) and SOF + LDV + GS-9451 (6 weeks), respectively. These results imply that the proportion of infectious virus drops rapidly over time at a rate that depends on the drug combination being used (Fig. 3), with about 0.1% and 0.05% of virus being infectious at W2 with dual and triple therapy, respectively. At W4, these proportions are even lower, with less than 0.01% and 0.003% of total circulating virus being infectious, respectively. These numbers vary according to the value assumed for *p*_0_ (Table 2). If one assumes that the proportion of infectious virus decreases immediately to a new fixed value, *p*_I_ after treatment initiation, the proportion of infectious virus had to drop to 0.00001% after treatment initiation to match the observed SVR, and this value is not sensitive to the pre-treatment value of *p*_0_ (Supplementary Text, Fig. S1 and Table S3).

**Table 2:** Minimal value of λ needed to predict 95% SVR after 8 weeks of treatment with SOF + LDV or after 6 weeks with triple therapy of SOF + LDV + GS-9669 or SOF + LDV + GS-9451 according to the proportion of non-infectious virus produced before treatment initiation.

Treatment	Duration (weeks)	Baseline proportion of infectious virus (%)	λ (day^−1^)	Predicted proportion of infectious virus (%)				
				W0	W1	W2	W4	W6
SOF + LDV	8	100	0.249	100	18.11	3.17	0.097	0.0030
		10	0.208	10	2.40	0.56	0.030	0.0017
		1	0.166	1	0.32	0.10	0.0098	0.00096
		0.1	0.125	0.1	0.042	0.018	0.0031	0.00053
SOF + LDV + GS-9669	6	100	0.357	100	8.63	0.071	0.0048	0.000032
		10	0.300	10	1.28	0.16	0.0023	0.000035
		1	0.247	1	0.18	0.033	0.0010	0.000032
		0.1	0.191	0.1	0.027	0.0071	0.00049	0.000034
SOF + LDV + GS-9451	6	100	0.327	100	10.61	1.075	0.011	0.00011
		10	0.270	10	1.57	0.24	0.0054	0.00012
		1	0.217	1	0.22	0.049	0.0024	0.00011
		0.1	0.162	0.1	0.033	0.011	0.0011	0.00011

Using the estimated value for λ for the different treatment regimens one can also predict the minimal SVR rate that can be expected for shorter treatment durations assuming that cure corresponds to the loss of the last infectious virion in the extracellular body fluids (see Methods). For SOF + LDV, we predict mean SVR rates after 6 weeks of treatment lower than 80% in all cases (Table 3). Likewise, for triple therapy, we predicted mean SVR rates after 4 weeks of treatment lower than 70% in all cases. On the contrary, using the model where effect of treatment on infectious virus is immediate, we predicted high SVR rates after short treatments (Table S4).

**Table 3:** The predicted SVR rates according to treatment duration and the proportion of non-infectious virus produced before treatment initiation. Prediction intervals were obtained by generating 1000 datasets of N = 50 patients. We assumed by construction that SVR rates of 95% were obtained after after 8 weeks of SOF+LDV or 6 weeks of SOF+LDV+GS-9669/GS-﻿9451﻿ (see Methods).

Treatment	Baseline proportion of infectious virus (%)	λ (day^−1^)	Predicted SVR rates for different treatment durations (%) Median (95% prediction interval)			
			3 weeks	4 weeks	6 weeks	8 weeks
SOF + LDV	100	0.249	2 (0-8)	14 (4–22)	60 (48–74)	95
	10	0.208	6 (0–12)	20 (10–30)	66 (52–80)	95
	1	0.166	10 (2–20)	28 (16–42)	72 (60–84)	95
	0.1	0.125	18 (8–30)	40 (26–54)	78 (64–90)	95
SOF + LDV + GS-9669	100	0.357	8 (2–16)	34 (22–50)	95	
	10	0.300	12 (4–22)	44 (30–58)	95	
	1	0.247	20 (10–32)	52 (38–66)	95	
	0.1	0.191	32 (20–46)	62 (48–76)	95	
SOF + LDV + GS-9451	100	0.327	14 (6–24)	44 (30–60)	95	
	10	0.270	20 (10–32)	52 (36–66)	95	
	1	0.217	30 (18–42)	60 (46–74)	95	
	0.1	0.162	42 (28–56)	68 (54–80)	95	

### Treatment individualization

Shortened treatment could still be relevant in patients if it is response-guided^12^. Thus, we stratified the SVR rate obtained above according to the early virologic response (viral load < 500 IU/mL at day 2 or < 12 IU/mL at day 7) with SOF + LDV + GS-9451 (Table 4). The model predicted that achieving a rapid virologic response at day 2 was not predictive of rapid viral eradication, with SVR rates in this population predicted to be lower than 66% and 92% (in the most favorable case considered) for treatment durations of 3 and 4 weeks, respectively. In contrast, undetectable viral load at week 1 was a more stringent but a better predictor of rapid viral eradication with a predicted SVR rate larger than 94% after 4 weeks of treatment in all cases considered.

**Table 4:** Predicted SVR rates according to the viral load at day 2 and the proportion of non-infectious virus produced before treatment initiation. Prediction intervals were obtained by generating 1000 datasets of N = 50 patients.

Treatment	Baseline proportion of infectious virus (%)	λ (day^−1^)	% patients with VL ≤ 500 IU/mL at day 2	Predicted SVR rates for different treatment durations(%) Median (95% prediction interval)			
				3 weeks in patients with VL > 500 IU/mL at day 2	3 weeks in patients with VL ≤ 500 IU/mL at day 2	4 weeks in patients with VL > 500 IU/mL at day 2	4 weeks in patients with VL ≤ 500 IU/mL at day 2
SOF + LDV + GS-9451	100	0.327	46.9	4 (0–12)	24 (12–36)	26 (14–40)	66 (52–88)
	10	0.270	46.9	8 (2–16)	34 (22–48)	32 (20–46)	74 (62–86)
	1	0.217	46.9	12 (4–22)	50 (36–64)	40 (26–52)	84 (74–92)
	0.1	0.162	46.9	20 (8–30)	66 (52–78)	34 (48–60)	92 (82–98)
**Treatment**	**Baseline proportion of infectious virus (%)**	**λ (day** ^**−1**^ **)**	**% patients with VL < 12 IU/mL at day 7**	**Predicted SVR rates for different treatment durations(%) Median (95% prediction interval)**			
				**3 weeks in patients with VL > 12 IU/mL at day 7**	**3 weeks in patients with VL ≤ 12 IU/mL at day 7**	**4 weeks in patients with VL > 12 IU/mL at day 7**	**4 weeks in patients with VL < 12 IU/mL at day 7**
SOF + LDV + GS-9451	100	0.327	23.8	4 (0–8)	50 (34–62)	30 (16–42)	94 (86–100)
	10	0.270	23.8	6 (0–14)	66 (52–78)	36 (24–50)	98 (92–100)
	1	0.217	23.8	12 (4–24)	82 (72–92)	48 (34–60)	100 (98–100)
	0.1	0.162	23.8	24 (14–36)	96 (90–100)	58 (44–70)	100 (100–100)

## Discussion

In the current era of highly effective treatments for HCV, there is a growing temptation to reduce treatment duration from 12 to 8, 6, 4 or even 3 weeks in order to improve adherence and reduce cost. Here our goal was to characterize the viral kinetics observed in two SOF-containing triple therapies used for 6 weeks. Based on experience accumulated with other anti-HCV drugs, our hypothesis was that in order to have a cure rate of 95% or higher in 6 weeks, the viral decline should be faster than seen with previous therapies. Further, by analyzing which aspects of viral kinetics were accelerated in triple therapies as compared to single or dual SOF-containing therapy, we expected that the effect of drug combinations could be better understood and help guide shorter treatment durations.

The viral kinetics that we observed were unexpected; the rate of viral decline after day 3 was no faster with triple SOF-containing therapies as compared to SOF + RBV only, suggesting that SOF + RBV had the same overall effect on HCV RNA kinetics as the other more potent combinations studied here. The decline observed in all groups was rather slow, with a final phase of viral decline rate equal to 0.14–0.20  day^−1^, consistent with the value found in HCV-HIV infected patients treated for 12 weeks with SOF + LDV (0.13–0.17 day^−1^ SVR rate of 98%)^13^. Larger values were reported with other SOF combinations^14, 15^. This difference could be due to different kinetic profiles but it could reflect the influence of the assay used for HCV RNA measurements on viral kinetic estimates. Here, we relied only on the Abbott real time HCV assay, which is generally considered as more sensitive and has smaller lower limit of detection than the Roche Taqman assay^16, 17^. Consequently, viral load may remain detectable for longer period of time which leads to lower estimates for the rate of viral decline during the terminal phase of the viral kinetics, i.e., the loss rate of infected cells.

Another very surprising observation was that 35% of patients treated for 12 weeks and as many as 55% treated for 6 weeks had detectable HCV RNA levels at EOT. Having HCV RNA close to the detection limit of 3 IU/mL implies that viral eradication is still far off. If about 10^12^ virions are produced daily in a typical patient to maintain a viral concentration of 10^6^ copies/mL^18^, then about 10^6^ virions need to be produced each day to obtain a viral concentrations of about 1 IU/mL. Consequently, one would expect that, if viremia is still detectable at the time of treatment cessation, then it should progressively rebound as active intracellular drug decays after the EOT. But in the two triple DAA arms of SYNERGY only one patient rebounded. This is not an isolated finding. For instance, in a cohort of 217 patients that received SOF + DCV or LDV for 12 weeks, 25% (53/217) had detectable HCV RNA at end of treatment, and nonetheless the SVR rate in this subgroup was about 95%^19^.

Three obvious possibilities can explain this phenomenon. First, this could be due to a long-lasting effect of SOF-containing combinations. However, our calculations showed that, assuming a constant antiviral effectiveness and the same kinetics of viral decline after EOT as on treatment, it would take more than 12 additional weeks in order to reach the cure boundary, i.e., well beyond the intracellular half-life of SOF^20^ and of LDV^21^. Second, chronic infection with HCV is associated with impaired immune function, characterized by progressive T-cell exhaustion^22^. This immune dysregulation was shown to be restored as a consequence of rapid viral clearance in patients successfully treated with all oral DAA therapies^23–25^. The restoration of the immune system may lead to a functional cure at higher viral loads than the theoretical cure boundary^26^. However, if this lack of viral rebound were immune system dependent, one would expect to observe more variability in the outcome, whereas here all but one patient achieved SVR. Lastly, even if pharmacological and immunological processes act in concert, we hypothesized that the most determinant factor explaining SVR in spite of having detectable HCV RNA at EOT was that the HCV RNA was essentially non-infectious.

This possibility is supported by the finding that both the NS5A and NS3 proteins play an important role in the production of infectious virus^27–30^ and that certain mutations in either the NS5A or NS3 gene can produce high proportions of non-infectious virus^27–31^. This suggests that a modification of NS5A or NS3 proteins, possibly due to drug interactions with these proteins or drug-induced changes in the vRNA replication machinery, could alter their roles in the assembly/secretion process, leading to the formation of non-infectious virus. In our extended multiscale model we assumed that the probability of secreting an infectious virion declined exponentially after treatment initiation with rate λ and found that if about 1% of virus is infectious before therapy^32, 33^ then λ had to be larger than 0.17, 0.25 and 0.22 day^−1^ to have all infectious virus cleared at EOT after 8 weeks of SOF + LDV, 6 weeks of SOF + LDV + GS-9669 and 6 weeks of SOF + LDV + GS-9451, respectively. This may indicate that not only NS5A inhibitors, but also non-nucleoside polymerase and protease inhibitors, increased the production of noninfectious virus. This result was confirmed by an *in vitro* study showing a significant loss of viral infectivity after treatment with NS5A inhibitors, in particular LDV and DSV, and to a lesser extent with a protease inhibitor^10, 34^. Consequently, our results suggest that total HCV RNA, particularly at EOT, may no longer be predictive of the outcome of these treatments and could be misleading by largely underestimating the effectiveness of therapy.

There are some limitations of our model. First, we used an approximate solution of the multiscale model where we neglected the generation of newly infected cells after treatment initiation^35^. Although this assumption is reasonable given the high treatment efficacy, it could nonetheless influence our estimate of λ. Second, the model assumes that a patient is cured once the predicted concentration of infectious virus has crossed the “cure boundary”, even though production of (defective) virus is still ongoing at and after EOT. Thus, the model assumes that infected cells still remaining at the end of treatment would continue to produce exclusively non-infectious virus after the end of treatment. In fact some patients had detectable HCV RNA 2–4 weeks after treatment cessation, implying that large quantities of virus continued to be produced after EOT. The fact that these newly produced virions do not rekindle infection suggests that the existing replication complexes may have been irreversibly damaged by the treatment and that infectious virus, if produced, is less fit or that immune system is sufficiently restored to control the infection. Third, the parameter λ was not estimated but rather was calibrated such that a SVR rate of 95% was achieved for 6-week treatment with triple DAA therapies and for 8-week SOF + LDV treatment, respectively. Of note lower SVR rates (82–95%) were observed in the TRIOLOGY-1 and TRIOLOGY-2 studies in which the same combinations were given for 8 weeks, but the patients were all cirrhotic^36^, whereas in the SYNERGY trial only 32% of patients had stage F3/4 fibrosis. This observation suggests that the treatment may have different effects in patients with and without cirrhosis.

The model predicted a mean SVR rate of 60–78% after 6-week treatment with SOF + LDV depending on the initial proportion of infectious virus, in line with the 68% of SVR found in the ELECTRON trial^5^. The model also predicted that 4 weeks of triple therapy would be suboptimal and would lead to a mean SVR rate of 34–68%. This is consistent with the SVR rate of 40% observed in patients receiving SOF + LDV + GS-9451 for 4 weeks in the second part of the SYNERGY trial^8^ and with the SVR rate of 39% observed in patients receiving SOF + elbasvir + grazoprevir for 4 weeks in the C-SWIFT study^7^. It also predicted a very suboptimal response in most patients treated for 3 weeks. However, these data were limited to small number of patients. With no phase 3 trials presently investigating treatments shorter than 8 weeks, it is difficult to confirm the predictions of our model in larger population.

Finally, we investigated the possibility of using this extended model to identify patients eligible for shorter treatment. Our model was stimulated by a recent study where all patients with an ultra-rapid response (viral load < 500 IU/mL at day 2) were cured after only 3 weeks of triple DAA treatment^12^. When we applied the same criterion, our model predicted a SVR rate lower than 70% in all cases considered (Table 4). However, all patients in this study were Chinese, infected with HCV genotype 1b, without cirrhosis and 85% had the IL28B CC genotype, which are predictive factors for SVR. On the contrary, the patients in the SYNERGY study used to construct our model were mostly of African descent, infected by HCV genotype 1a, 32% with F3-4 fibrosis and only 20% had the IL28B CC genotype (Table S1), which are unfavorable factors for SVR. Therefore, the predictions of our model should not be generalized to other populations. For patients having the same characteristics as the patients in the SYNERGY trial, the model predicted that 4 week treatments may be sufficient in those having undetectable viremia at week 1 with SVR rates larger than 94%. This criterion could be relevant to test in future studies, but it is probably too conservative as many individuals with detectable viremia at week 1 could still go on to obtain SVR. In fact, in the different scenarios that we considered, 30–60% of patients with detectable viremia at week 1 were predicted to nonetheless achieve SVR within 4 weeks. That represents 32–48% of patients that were predicted to be cured in four weeks. In other words, this criterion is not very sensitive and a large proportion of patients could eradicate virus in less than 4 weeks in spite of having detectable viremia by week 1. Therefore, the benefit of response-guided treatment might be limited in the context of these very short treatments. Also, in the Chinese study discussed above, the patients who did not achieve a viral load <500 IU/mL by day 2 were switched to standard SOF + LDV therapy for 12 weeks so it is hard to say if response guided therapy was needed to achieve SVR by three weeks even in this population.

In conclusion, we have shown that the kinetics of viral decline in the SYNERGY trial patients was remarkably slow, which is paradoxical given that at least 95% of patients treated for 6 weeks achieved SVR. We proposed a model in which noninfectious virus was generated during the assembly and secretion process to resolve this paradox and we showed that this model could recapitulate the observed clinical results. Whatever the mechanisms involved in this slow viral decline our results suggest that, unlike what was known for previous anti-HCV treatments, relying on HCV RNA kinetics during treatment or even simply on the viral load level at the end of treatment may no longer be relevant to predict the outcome of SOF + LDV containing regimens. Our model shows that the monitoring of the early virologic response may be still beneficial to some patients in order to reduce treatment duration. However new criteria for response guided therapy will have to be defined rigorously in adequately-powered and randomized clinical studies, and probably will need to rely on markers in addition to viral load.

## Materials and Methods

### Patients and study design

We used data from the SYNERGY and SPARE trials (ClinicalTrials.gov, numbers NCT01805882 and NCT01441180, respectively)^6, 37^. Written or oral informed consent from all participants was obtained. The clinical studies were approved by the institutional review board of the National Institute of Allergy and Infectious Diseases (NIAID) and were done in compliance with the Good Clinical Practice guidelines, the Declaration of Helsinki, and regulatory requirements. More details can be found in the primary papers^6, 37^.

The SPARE trial was conducted in two parts. In the first part, patients with a moderate stage of liver fibrosis were treated for 24 weeks with SOF (400 mg/day) once a day and a weight-based RBV regimen. In the second part, patients with all stages of liver fibrosis were randomized to receive the same dose of SOF in combination with either weight-based or low-dose RBV (600 mg/day) for 24 weeks^37^.

In the SYNERGY trial, patients were randomized into three groups. Patients with or without cirrhosis in the first group were given once a day SOF (400 mg) and LDV (90 mg) for 12 weeks. In the two remaining groups, patients without cirrhosis were treated for only 6 weeks and received GS-9669 (250 mg twice a day) or GS-9451 (80 mg once a day) on top of SOF + LDV^6^.

Baseline characteristics were similar among different treatment groups (Table S1). Participants were all treatment naïve, predominantly black (about 80%), infected with HCV genotype 1a or 1b, had high baseline plasma HCV RNA levels >800,000 IU/mL (70%) and a stage of fibrosis of 0–2 (70%).

### Viral load data

Plasma HCV RNA was measured using the real-time HCV assay (Abbott Molecular), with a lower limit of quantification (LOQ) of 12 IU/mL and a lower limit of detection (LOD) of 3 IU/mL. Six patients in the SPARE trial were not included in the analysis: five had virological rebound due to non-adherence to treatment and one had a very low initial viral load (63 IU/mL). Using these data, the overall SVR rates were 69%, 100%, 95% and 95% with SOF + RBV, SOF + LDV, SOF + LDV + GS-9669 and SOF + LDV + GS-9451, respectively.

### Mathematical modeling

#### Multiscale model

The viral kinetics was characterized using the approximate solution of the multiscale model developed for DAAs^35^:

1 $$V(t)={V}_{0}\,({e}^{-ct}+(1-{\varepsilon }_{s})\frac{c\rho }{N}(\frac{A}{B\delta (\delta -c)}({e}^{-ct}-{e}^{-\delta t})+\frac{1}{B+\delta -c}(\frac{N}{\rho }-\frac{A}{B\delta })({e}^{-ct}-{e}^{-(B+\delta )t})))$$

where *V*_0_ is the baseline viral load, *V(t)* is the viral load after time *t* on therapy, *c* is the viral clearance rate, *δ* is the loss rate of infected cells, $A=(1-{\varepsilon }_{\alpha })\alpha$, $B=(1-{\varepsilon }_{s})\rho +\kappa \mu$ and $N=\frac{\rho (\alpha +\delta )}{\delta (\rho +\mu +\delta )}$. In this model, *α*, *ρ* and *μ* are the rates of viral RNA production, viral assembly/secretion and viral RNA (vRNA) degradation, respectively. Antiviral drugs can have an effect by blocking vRNA production with an effectiveness ε_α_, by blocking viral assembly/secretion with effectiveness ε_s_, where the effectiveness parameters are between 0 and 1, with 1 corresponding to 100% effectiveness. They might also enhance the intracellular viral degradation rate by a factor κ. For the sake of parameter identifiability we assumed no effect in enhancing the degradation rate of vRNA and we fixed κ to 1.

#### Extended multiscale model to incorporate the generation of non-infectious during treatment

Because the original multiscale model systematically under-predicted the observed SVR rate in the Synergy trial (see results) we modified the model by distinguishing infectious and non-infectious virus, denoted *V*_I_ and *V*_NI_, respectively. We let *p*_I_ be the proportion of vRNA assembled and packaged as infectious virus among the total virus released at a given time. We assumed that treatment gradually reduces the amount of infectious virus such that *p*_I_ is given by the following equation:

2 $${p}_{I}(t)={p}_{0}\ast {e}^{-{\rm{\lambda }}t}$$

where *p*_0_ is the proportion of infectious virus in absence of treatment, and λ is a parameter measuring the rate of decrease of this proportion over time after treatment initiation. Of note, this model assumes that the proportion of infectious virus tends to 0 as time proceeds.

The equation for the total virus, *V*=*V*_I_ + *V*_NI_ remains unchanged from that derived in Rong *et al*.^38^ and is given by Eq. 1, while the equation for the infectious virus *V*_I_ is given by (see Supplementary Text):

3 $$\begin{array}{rcl}{V}_{I} & = & {p}_{0}{V}_{0}({e}^{-ct}+(1-{\varepsilon }_{s})\frac{c\rho }{N}(\frac{A}{B\delta (\delta +\lambda -c)}({e}^{-ct}-{e}^{-(\delta +\lambda )t})\\  &  & +\frac{1}{B+\delta +\lambda -c}(\frac{N}{\rho }-\frac{A}{B\delta })({e}^{-ct}-{e}^{-(B+\lambda +\delta )t})))\end{array}$$

Another model was also tested where ${p}_{I}(t)={p}_{1}\ne 0$ for t > 0, i.e., the effect of treatment in generating noninfectious virus was constant and immediate after treatment initiation (see supplementary Text).

#### Estimation of viral kinetic parameters and effects of treatment

Although patients in the SPARE trial treated with the highest dose of RBV had significantly higher SVR rates than patients treated with a lower dose, no difference in viral kinetics during treatment was observed^37^ and therefore we considered the Spare trial as a single treatment group. Thus four treatment groups with different treatment durations were considered: SOF + RBV (24 weeks), SOF + LDV (12 weeks), SOF + LDV + GS-9669 (6 weeks) and SOF + LDV + GS-9451 (6 weeks). Only viral load data during treatment were included in the data fitting. Because there were large differences in treatment duration, which could possibly induce bias in the parameter estimation, we verified that our estimates remained unchanged when using only data obtained during the first 7, 14 or 42 days of treatment (not shown).

Because the solution for the total viral load of the extended model is the same as in the original multiscale model, all viral kinetic parameters except *p*_0_ and λ (see below) were estimated by fitting the observed viral load to Eq. 1. Differences across the treatment groups in the following parameters were tested: ε_s_ (effect in blocking viral assembly/secretion), ε_α_ (effect in blocking viral RNA production) and δ (loss rate of infected cells). All drugs block viral RNA production but only NS5A and protease inhibitors block viral assembly and secretion, therefore we fixed ε_s_ = 0 in patients that received SOF + RBV^14^. All treatment group effects were assessed by covariate testing using the likelihood ratio test (LRT). Viral kinetic parameters and their inter-individual variability were estimated in MONOLIX 4.3 using the SAEM algorithm to take into account data below the limit of quantification and detection.

#### Estimation of the proportion of infectious virus over time

Unlike other viral kinetic parameters, the two additional parameters of the extended model, *p*_0_ and λ, cannot be directly estimated from viral load data because they do not impact the total virus concentration, which is the only quantity that can be observed. For *p*_0_, a parameter independent of treatment, *in vitro* experiments suggest that it could range from 0.1% to as high as 100%^32, 33^ and that *in vitro* titers could be correlated with the *in vivo* infectivity^39^. Therefore we fixed *p*_0_ at different values within the range from 0.1% to 100%.

For each fixed value of *p*_0_, we then found the decay rate of the infectious proportion over time, λ, as follows. For each treatment group of the SYNERGY trial, 1,000 *in silico* patients were generated using the viral kinetic parameters and their distribution (see Table 1) estimated from the total viral load and we calculated for each patient the minimal value for λ such that all infectious virus particles are below the “cure boundary” (see below) at the end of 8 weeks of SOF + LDV or 6 weeks of SOF + LDV + GS-9451 or SOF + LDV + GS-9669. The value for λ that was retained was the 95^th^ percentile of the distribution, such that by construction eradication of infectious virus is obtained in each treatment group in 95% of patients at the end of treatment (12 weeks for SOF + LDV, 6 weeks for SOF + LDV + GS-9451 or SOF + LDV + GS-9669), as observed in the SYNERGY trial. Of note, since data from a phase 3 study showed significant non-inferiority of a treatment duration with SOF + LDV of 8 vs 12 weeks and that 8-week treatment had SVR rates of 94% in both groups^4^, we took 8 weeks of treatment as the reference treatment duration with SOF + LDV.

#### Predicting SVR rates from the viral kinetics observed during treatment

To predict whether a patient will achieve SVR, we used the notion of a “cure boundary”, a theoretical value defined as having less than one virus particle in the total body extracellular fluid, corresponding to a viral concentration of 6.7 × 10^−5^ IU/mL^40^. If the predicted patient’s viral load at the end of treatment was below this level, we assumed that SVR will be achieved. For the original multiscale model (Eq. 1) which does not distinguish between infectious and non-infectious virus, viral eradication is based on the predicted total viremia, while in the extended multiscale model (Eq. 1) it is defined using the predicted infectious virus concentration (Eq. 3).

The SVR rate of a treatment group was predicted using viral kinetic profiles of 50,000 *in silico* patients, simulated from the model parameter estimates obtained in the previous steps. In order to account for the variability that can be observed in small size clinical trials, a 95% prediction interval for the SVR rate was calculated assuming 1000 *in silico* trials of 50 patients each.

## Electronic supplementary material

Supplementary materials
