# Supplementary material for: The paradox of highly effective sofosbuvir-based combination therapy despite slow viral decline: can we still rely on viral kinetics?
Source: Sci Rep. 2017 Aug 31;7:10233. doi: 10.1038/s41598-017-09776-z (PMC5579268; doi:10.1038/s41598-017-09776-z)
Supplement: Supplementary file 1 — Supplementary materials [file 41598_2017_9776_MOESM1_ESM.pdf]

The paradox of highly effective sofosbuvir-based combination therapy despite slow viral decline: can we still rely on viral kinetics?

Thi Huyen Tram Nguyen[1], Jérémie Guedj[1,2], Susan L. Uprichard[3], Anita Kohli[4], Shyam Kottilil[5], Alan S Perelson[6]

Supplementary Tables

Table S1. Patient baseline characteristics in the various treatment groups

	SOF + LDV (N=20)	SOF + LDV + GS-9669 (N=20)	SOF + LDV + GS-9451 (N=20)	SOF + Weight-based RBV (Part 1) (N=10)	SOF Weight-based RBV (Part 2) (N=25)	SOF Low-dose RBV (Part 2) (N=25)
**Sex (% male)**	70	65	80	40	76	40
**Body mass index* (kg/m2)**	25 (23-28)	27 (24-29)	29 (25-31)	26 (26.0-34.0)	28 (25-31)	30 (27-37)
**Body mass index ≥ 30**	3	6	9	3	12	14
**Genotypes (GT)**						
**1a**	11	14	17	6	20	16
**1b**	9	6	3	4	5	9
**HCV RNA* (log_{10}IU/mL)**	6.3 (5.9-6.5)	6.1 (5.8-6.4)	6.2 (5.8-6.4)	6.8 (6.0-7.1)	6.2 (5.4-6.4)	6.1 (5.5-6.3)
**HCV RNA>800 000 IU/mL**	15	13	14			
**HAI Fibrosis**						
**0-2**	12	15	14	9	9	9
**3-4**	8	5	6	1	1	1
**IL28B**						
**CC**	5	2	5	3	4	4
**CT/TT**	15	18	15	6	21	21
**IFNL4**						
**TT/TT**	3	3	5			
**dG/TT**	10	10	6			
**dG/dG**	7	7	9			
**Ethnic**						
**Black**	18	19	18	9	18	23
**White**	2	1	2	1	5	2
**Hispanic**	0	0	0	0	2	0

*Median (min-max)

Table S2. The predicted SVR rates (%) and the 95% prediction interval (N=50 patients) obtained via 1000 simulations and the observed SVR rate in different treatment arms of the SPARE and SYNERGY studies. In bold are the predicted SVR rates for the treatment duration given in the SYNERGY and SPARE trials. The observed SVR rate is the SVR rate reported in these studies.

	W6	W8	W12	W16	W20	W24	Observed SVR
**SOF + RBV**	16 (6-26)	28 (16-42)	54 (40-66)	72 (56-82)	82 (70-92)	**90 (78-96)**	**48-68 (24 weeks)**
**SOF + LDV**	6 (0-14)	14 (6-24)	**34 (20-46)**	52 (38-64)	66 (52-78)	76 (62-86)	**100 (12 weeks)**
**SOF + LDV + GS-9669**	**6 (0-14)**	14 (6-24)	34 (20-46)	52 (38-64)	66 (52-78)	76 (62-86)	**95 (6 weeks)**
**SOF + LDV + GS-9451**	**16 (6-26)**	30 (18-42)	54 (40-68)	72 (58-84)	82 (72-92)	90 (80-96)	**100 (6 weeks)**

Table S3. Minimal value of p_I needed to predict 95% SVR after 8 weeks of treatment with SOF+LDV or after 6 weeks with triple therapy of SOF+LDV+GS-9669 or SOF+LDV+GS-9451 using the second model assuming an immediate and constant effect of treatment on infectious virus. The value estimated for p_I in this model does not depend on the initial value of p_0

**Treatment**	**Duration (weeks)**	p_I
SOF+LDV	8	9.5×10^{-7}
SOF+LDV+GS-9669	6	3.5×10^{-7}
SOF+LDV+GS-9451	6	1.2×10^{-6}

Table S4. The SVR rates (%) and the 95% prediction interval (N=50) obtained via 1000 simulations predicted with the second model assuming an immediate and constant effect of treatment on infectious virus and the observed SVR for different combination and treatment duration

Treatment	p_I	Predicted SVR rate for different treatment duration* (%)		
		**4 weeks**	**6 weeks**	**8 weeks**
SOF+LDV	9.5×10^{-7}	80 (68-92)	90 (80-98)	95
SOF+LDV+GS-9669	3.5×10^{-7}	90 (78-96)	95	
SOF+LDV+GS-9451	1.2×10^{-6}	88 (76-96)	95	

*Median (95% prediction interval for a cohort of 50 patients)

Supplementary Text

In order to capture the profound first phase decline observed in the Synergy trial, we used the multiscale model developed by Rong et al. [1] to analyze the viral load data. Here we extended the model to account for infectious and noninfectious virus:

$$\frac{d}{dt}T(t) = s - \beta V_i(t)T(t) - dT(t) \tag{1}$$
$$\frac{\partial}{\partial t}I(a,t) + \frac{\partial}{\partial a}I(a,t) = -\delta I(a,t)$$
$$I(0,t) = \beta V_i(t)T(t), \quad I(a,0) = \bar{I}(a)$$
$$\frac{\partial}{\partial t}R(a,t) + \frac{\partial}{\partial a}R(a,t) = (1-\varepsilon_\alpha)\alpha - [(1-\varepsilon_s)\rho + \kappa\mu]R(a,t)$$
$$R(0,t) = 1, \qquad R(a,0) = \bar{R}(a)$$
$$\frac{d}{dt}V_i(t) = (1-\varepsilon_s)p_i(t)\int_0^\infty \rho R(a,t)I(a,t)da - cV_i(t)$$
$$\frac{d}{dt}V_{ni}(t) = (1-\varepsilon_s)(1-p_i(t))\int_0^\infty \rho R(a,t)I(a,t)da - cV_{ni}(t)$$

where a is the infection age and α, ρ, μ are constant rates of vRNA production, assembly/secretion and degradation, respectively. $\bar{I}(a)$, $\bar{R}(a)$ are the pretreatment steady-state distributions of the infected cells and intracellular vRNA, respectively. In this model, drugs can have antiviral effect via: i) blocking vRNA production with effectiveness ε_α, ii) blocking viral assembly/secretion with effectiveness ε_s, iii) enhancing the production of noninfectious virus, i.e. by decreasing p_i, *iv)* enhancing the degradation rate of vRNA by a factor κ.

We do not present step-by-step solution for this model here. For more details, see the paper of Rong et al.[1]. At the steady state before treatment initiation, $\varepsilon_\alpha = \varepsilon_s = 0$ and $p_i = p_0$. Integrating the R and I equations in the pre-therapy model, we get the solutions for $R(a,t)$ and $I(a,t)$. The equation for $R(a,t)$ remains unchanged as compared to the initial model (without infectious virus) (16), while the solution for $I(a,t)$ is given by:

$$I = \begin{cases} \beta V_i(t-a)T(t-a)e^{-\delta a} & for\ a < t \\ I_0(a-t)e^{-\delta t} & for\ a \geq t \end{cases} \tag{2}$$

At the pre-therapy steady state, $\frac{d}{dt}V_i(t) = 0$, therefore

$$p_0\int_0^\infty \rho\bar{R}(a)\bar{I}(a)da - c\bar{V}_i = 0 \tag{3}$$

where the overbars denote steady state quantities.

At steady state $\bar{I}(a) = \beta\bar{V}_i\bar{T}e^{-\delta a}$, and thus we obtain

$$p_0 \int_0^\infty \rho\bar{R}(a)\beta\bar{V}_i\bar{T}e^{-\delta a}da - c\bar{V}_i = 0 \tag{4}$$

Hence

$$\beta\bar{V}_i\bar{T} = \frac{c\bar{V}_i}{p_0\int_0^\infty \rho\bar{R}(a)e^{-\delta a}da} = \frac{c\bar{V}_i}{p_0 N} \tag{5}$$

where $N = \int_0^\infty \rho\bar{R}(a)e^{-\delta a}da = \frac{\rho(\alpha+\delta)}{\delta(\rho+\mu+\delta)}$.

After initiation of treatment at time t=0, $\varepsilon_\alpha \neq 0$, $\varepsilon_s \neq 0$ and $p_i \neq p_0$. Here, we assumed that the treatment effect reached very high values after the first doses of antivirals (hence, little change in drug efficacy during treatment) and that few new infections could occur after treatment initiation, i.e., $I(a,t) = R(a,t) = 0$ for $a < t$. Under this assumption, the equation for $R(a,t)$ can be described by:

$$R(a,t) = \frac{A}{B} + \left(\bar{R}(a-t) - \frac{A}{B}\right)e^{-Bt} \quad for\ a \geq t \tag{6}$$

where $A = (1-\varepsilon_\alpha)\alpha$, $B = (1-\varepsilon_s)\rho + \kappa\mu$.

Using (2), (5), and (6), we have

$$\int_0^\infty R(a,t)I(a,t)da = \int_0^\infty \left(\frac{A}{B} + \left(\bar{R}(a-t) - \frac{A}{B}\right)e^{-Bt}\right)\beta\bar{V}_i\bar{T}e^{-\delta a}da \tag{7}$$

$$= \beta\bar{V}_i\bar{T}\int_0^\infty \left(\frac{A}{B} + \left(\frac{\alpha}{\rho+\mu} + \left(1 - \frac{\alpha}{\rho+\mu}\right)e^{-(\rho+\mu)(a-t)} - \frac{A}{B}\right)e^{-Bt}\right)e^{-\delta a}da$$

$$= \beta\bar{V}_i\bar{T}\left(\frac{A}{B\delta}e^{-\delta t} + \left(\frac{\alpha+\delta}{\delta(\rho+\mu+\delta)} - \frac{A}{B\delta}\right)e^{-(B+\delta)t}\right)$$

$$= \frac{c\bar{V}_i}{p_0 N}\left(\frac{A}{B\delta}e^{-\delta t} + \left(\frac{N}{\rho} - \frac{A}{B\delta}\right)e^{-(B+\delta)t}\right)$$

Plugging (7) into the equation for $V_i(t)$, we obtain

$$\frac{d}{dt}V_i(t) = (1-\varepsilon_s)p_i(t)\rho\int_0^\infty R(a,t)I(a,t)da - cV_i(t) \tag{8}$$

$$= (1-\varepsilon_s)p_i(t)\rho\frac{c\bar{V}_i}{p_0 N}\left(\frac{A}{B\delta}e^{-\delta t} + \left(\frac{N}{\rho} - \frac{A}{B\delta}\right)e^{-(B+\delta)t}\right) - cV_i(t)$$

Solution for Model 1.

For Model 1, we have

$$p_i(t) = p_0 e^{-\lambda t} \tag{9}$$

Therefore:

$$\frac{d}{dt}V_i(t) = (1-\varepsilon_s)e^{-\lambda t}\rho\frac{c\bar{V}_i}{N}\left(\frac{A}{B\delta}e^{-\delta t} + \left(\frac{N}{\rho} - \frac{A}{B\delta}\right)e^{-(B+\delta)t}\right) - cV_i(t) \tag{10}$$

Solving equation (10), where $\bar{V}_i = p_0 V_0$, the baseline level of infectious virus, we obtain:

$$V_i(t) = p_0 V_0 \Bigg(e^{-ct} + (1-\varepsilon_s)\frac{c\rho}{N}\left(\frac{A}{B\delta(\delta+\lambda-c)}\left(e^{-ct} - e^{-(\delta+\lambda)t}\right) + \frac{1}{B+\delta+\lambda-c}\left(\frac{N}{\rho} - \frac{A}{B\delta}\right)\left(e^{-ct} - e^{-(B+\lambda+\delta)t}\right)\right)\Bigg) \tag{11}$$

Solution for Model 2.

For Model 2, we have:

$$p_i(t) = \begin{cases} p_0 & t \leq 0 \\ p_I & t > 0 \end{cases} \tag{12}$$

Therefore, for $t > 0$,

$$\frac{d}{dt}V_i(t) = (1-\varepsilon_s)p_I\rho\frac{c\bar{V}_i}{p_0 N}\left(\frac{A}{B\delta}e^{-\delta t} + \left(\frac{N}{\rho} - \frac{A}{B\delta}\right)e^{-(B+\delta)t}\right) - cV(t) \tag{13}$$

Solving equation (13), we obtain:

$$V_I = V_0 p_0 \left(e^{-ct} + (1-\varepsilon_s)\frac{p_I c\rho}{p_0 N}\left(\frac{A}{B\delta(\delta-c)}\left(e^{-ct} - e^{-\delta t}\right) + \frac{1}{B+\delta-c}\left(\frac{N}{\rho} - \frac{A}{B\delta}\right)\left(e^{-ct} - e^{-(B+\delta)t}\right)\right)\right) \tag{14}$$

where $A = (1-\varepsilon_\alpha)\alpha$, $B = (1-\varepsilon_s)\rho + \kappa\mu$ and $N = \frac{\rho(\alpha+\delta)}{\delta(\rho+\mu+\delta)}$.

Model 2: Treatment effect increased abruptly after treatment initiation

In this model we assumed that the proportion of infectious virus packaged and released increased to its maximum value, $p_I > 0$, immediately after treatment initiation., Like in Model 1, the equation for the total virus remains unchanged, however the concentration of infectious virus is given by equation (14).

In order to estimate p_I, we generated 1,000 in silico patients using the distribution of parameter values found with the initial model. Then, for each patient, we calculated the minimal value of p_I leading to a SVR with 8 or 6 weeks of treatment with SOF+LDV or SOF+LDV+GS-945/GS-9669, respectively.

Then we estimated p_I as the value leading to a SVR rate of 95% after 8 weeks of treatment with SOF+LDV or 6 weeks of treatment SOF+LDV+GS-9451/GS-9669.

Unsurprisingly, the estimated value of p_I was very close to 0 for the three combinations in the Synergy trial: only 10, 4 and 12 per 10^7 newly produced virions are infectious with SOF+LDV, SOF/LDV/GS-9669 and SOF/LDV/GS-9451, respectively (**Table S3**). This model predicts an extremely rapid drop of infectious virus: after one day of treatment, the fraction of infectious virus is already lower than 1% (~0.07%) in all treatment arms. This is because in this model, the effect of treatment is carried in the first phase of viral load decline. After this first phase decline, the infectious and noninfectious virus will decline at the same rate (**Fig S1**). Therefore, this effect of treatment should be extremely high to attain rapidly a proportion of infectious virus, which is low enough to cure patients at EOT. The prediction of the SVR rate with different treatment durations obtained with this model is given in **Table S4**. In contrast to the model presented in the main text, this model predicts high SVR rates for short treatment durations. The initial proportion of infectious virus does not influence the estimation of p_I and the predictions of this model.

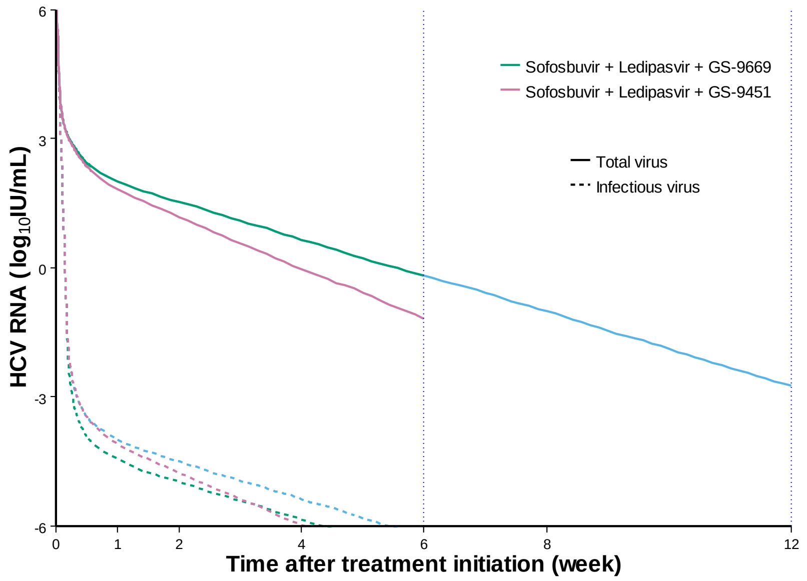

Figure S1. The predicted total and infectious virus using median parameter values with Model 2 (Initial proportion of infectious virus was set at 100%)

References:

1. Rong, L. *et al.* Analysis of hepatitis C virus decline during treatment with the protease inhibitor danoprevir using a multiscale model. *PLoS Comput. Biol.* **9,** e1002959 (2013).
